# Neuromuscular manifestations of wild type transthyretin amyloidosis: a review and single center’s experience

**DOI:** 10.3389/fcvm.2024.1345608

**Published:** 2024-02-12

**Authors:** Sasha A. Živković, David Lacomis, Prem Soman

**Affiliations:** ^1^ Department of Neurology, Yale University, New Haven, CT, United States; ^2^ Department of Neurology, University of Pittsburgh Medical Center, Pittsburgh, PA, United States; ^3^ Cardiac Amyloidosis Center, UPMC Heart and Vascular Institute, Pittsburgh, PA, United States; ^4^ Department of Pathology (Neuropathology), University of Pittsburgh Medical Center, Pittsburgh, PA, United States; ^5^ Division of Cardiology, University of Pittsburgh Medical Center, Pittsburgh, PA, United States

**Keywords:** wild type amyloidosis, transthyretin amyloidosis (ATTR), peripheral neuropathy (PN), carpal tunnel syndrome, dysautonomia, musculoskeletal disorders (MSD)

## Abstract

Transthyretin amyloidosis (ATTR) is a condition defined by accumulation of insoluble transthyretin amyloid deposits in multiple organs, especially in the peripheral nerve and heart muscle. ATTR may result from transthyretin mutations (variant ATTR or ATTRv) or may occur with normal transthyretin genotype (wild type ATTR or ATTRwt). ATTRwt was previously known as “senile amyloidosis” and causes cardiomyopathy which may lead to heart failure with a preserved ejection fraction, affecting predominantly elderly men. The exact prevalence of ATTRwt in the general population remains unclear, but its occurrence may be underestimated in women. It was observed that a proportion of ATTRwt cardiomyopathy patients may develop slowly progressing neuropathy that is milder and indolent in comparison with typical progressive neuropathy associated with ATTRv. Furthermore, the causality of neuropathy is often uncertain in patients with ATTRwt. Neuropathy symptoms, including distal sensory loss, unsteadiness and (neuropathic) pain are common in elderly patients with multiple potential causes, and as ATTRwt patients are typically older, relatively high prevalence of peripheral neuropathy is expected with frequent comorbidities. Relatively high prevalence of ATTRwt in elderly population contrasts few documented cases of neuropathy caused by ATTRwt, and there is uncertainty whether ATTRwt neuropathy is an infrequent occurrence or a significant manifestation of multisystemic ATTRwt. We review neurologic and musculoskeletal manifestations of ATTRwt and present clinical features of a single center cohort of ATTRwt patients with suspected peripheral neuropathy.

## 1 Introduction

**Figure 1:**
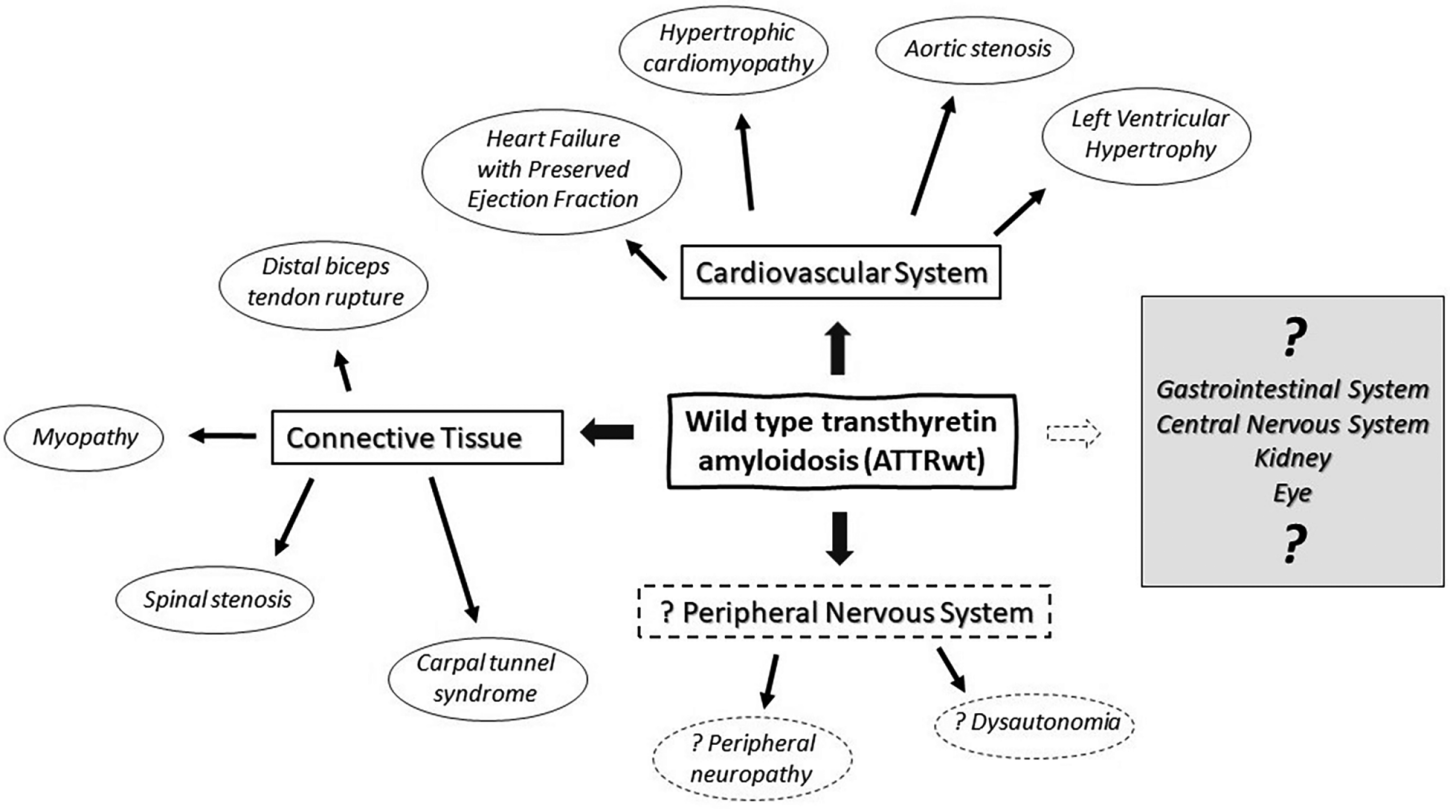
Multisystemic manifestations of wild type transthyretin amyloidosis (ATTRwt) in cardiovascular system, peripheral nervous system and connective tissue.

Transthyretin (TTR), also known as prealbumin, is an abundant plasma protein which shuttles vitamin A and thyroxine (1). Mutations of TTR can be associated with accumulation of insoluble transthyretin deposits in various organs and tissue causing hereditary or variant TTR amyloidosis (ATTRv) (2). In ATTRv, the phenotype is strongly influenced by genotype, although significant variation may exist within the same family. Two major phenotypes are cardiac and neuropathic, with many patients having some degree of mixed cardiomyopathy and neuropathy (3), and many other organs and tissue get affected as well. In individuals with a normal TTR genotype, insoluble amyloid deposits may also accumulate primarily in the heart and cause cardiomyopathy associated with wild type transthyretin amyloidosis (ATTRwt). ATTRwt was previously known as “senile amyloidosis” and is found in 13% of patients of heart failure with a preserved ejection fraction (4). Incidence of ATTRwt increases with aging and it typically affects elderly men (85%–91%) with a median age of 75–79 years and median survival of 3.6–4.9 years (4, 5). In addition to heart, ATTRwt deposition is found in connective tissue including forearm flexor, biceps brachii, rotator cuff tendons and in ligamentum flavum of patients with lumbar stenosis (Table 1) (6–9). The exact prevalence of ATTRwt in the general population remains unclear and the frequency of its occurrence may be underestimated in women. Deposits of ATTRwt were found in 25% of autopsies performed after age 85 in Finland (16). While peripheral and autonomic neuropathy are common in ATTRv, association with ATTRwt remains a matter of an ongoing debate. Neuromuscular symptoms, including distal sensory loss, unsteadiness and (neuropathic) pain are also common in elderly patients, and often there are multiple potential etiologies (17). It has been estimated that up to 13% of population older than 52 may have probable or definitive peripheral neuropathy, and the prevalence further increases with aging (17). As ATTRwt patients are typically older, relatively high prevalence of peripheral neuropathy is expected with frequent comorbidities (18, 19). It was observed that a proportion of ATTRwt cardiomyopathy patients may develop slowly progressing neuropathy that is milder and indolent in comparison with typical progressive neuropathy associated with ATTRv (18–24). Nevertheless, clinical significance of ATTRwt neuropathy is still not well understood, and the causality often remains unresolved in individual cases. Both ATTRv and ATTRwt are also associated with amyloid deposits in connective tissue with secondary neurologic manifestations and high prevalence of carpal tunnel syndrome, while spinal stenosis is also common in ATTRwt (7, 18, 19, 21–28). While ATTRwt deposits are found in muscle tendons and ligaments, heart muscle and other types of tissue (Figure 1) (29, 30), overall multisystemic manifestations of ATTRwt are not as prominent as in ATTRv (25).

**Table 1:** Neurologic and musculoskeletal distribution and clinical manifestations of amyloid deposition in wild type transthyretin amyloidosis (ATTRwt) (6–15).

	Location of ATTRwt deposits	Clinical manifestations
Peripheral nervous system	Peripheral nerve	Peripheral neuropathy
	Skeletal muscle	Myopathy
Musculoskeletal system	Muscle tendons	Biceps Brachii distal tendon rupture
	Wrist tenosynovium	Carpal tunnel syndrome
	Ligamentum flavum	Spinal stenosis (radiculopathy, myelopathy)
	Rotator cuff	? (currently unknown)
	Knee and hip synovium and cartilage	? (currently unknown)

We review neuromuscular manifestations of ATTRwt, discuss their clinical significance and present clinical features of a cohort of 18 ATTRwt patients with suspected peripheral neuropathy who were evaluated at UPMC Neuromuscular Clinic between 2017 and 2022.

## 2 Pathogenesis of ATTRv and ATTRwt

Physiologic role of TTR consists of shuttling thyroxine and retinol-binding protein bound to vitamin A, and it may also have neuroprotective properties (1). The pathogenesis of tissue tropism and damage in transthyretin amyloidosis (ATTR) is still not well understood, but it has been proposed that local features of endoplasmatic reticulum-assisted folding and degradation of TTR may determine tissue selectivity of amyloid accumulation (31). Two major targets for ATTR deposition in ATTRv are peripheral nerve and cardiac muscle. In the peripheral nerve, it is believed that an early disruption of blood-nerve barrier facilitates entry of circulating TTR and amyloid deposition (32). The exposure of Schwann cells and peripheral nerves to amyloid fibrils and non-fibrillar TTR may result in toxicity, and there may be a role for mechanical stress with amyloid fibril elongation (33). Similar changes were reported in the retinal and choroid vasculature, suggesting disruption of blood-retinal barrier that may promote ocular amyloid deposition (34). In the heart, cardiac muscle infiltration by ATTR deposits leads to stiffening and reduced contractility (35). The main mechanism of amyloid formation in ATTRv starts with dissociation of TTR tetramers and most of TTR pathogenic mutations result in a less stable TTR tetramer promoting its dissociation into monomers (2). Misfolding of monomers is then followed by oligomer aggregation and formation of full-length TTR amyloid fibrils (2). Low-grade dissociation and aggregation may occur physiologically, and may eventually lead to ATTRwt (36). Additionally, the other TTR amyloid pathway involves proteolytic cleavage leading to formation of carboxyterminal TTR fragments which are more prone to aggregation and amyloid formation with short amyloid fibrils. Proteolytic pathway may be relevant for ATTRwt and late onset ATTRv. Amyloid variant deposits typically have both types of amyloid fibrils with full-length amyloid fibrils found as predominant in early onset V30M ATTRv, and short amyloid fibrils in late-onset V30M ATTRv and in ATTRwt (37). In ATTRv, amyloid deposits often contain both ATTRv and ATTRwt (38). The clinical reports of biopsy-proven ATTRwt-associated neuropathy are exceedingly rare and at this time it remains uncertain whether ATTRwt is a significant cause of peripheral neuropathy in general population.

## 3 Methods

Our cohort included 18 consecutive patients with wild-type transthyretin amyloidosis who were evaluated for suspected peripheral neuropathy at UPMC Neuromuscular Clinic between 2017 and 2022. Clinical data was collected retrospectively, and the study was approved by University of Pittsburgh IRB. The diagnosis of “ATTRwt with neuropathy” was defined as ATTRwt proven by imaging and/or histopathology with concurrent clinical suspicion of peripheral neuropathy. Our subjects were predominantly men (95%) with a mean age of 72.2 years at the time of diagnosis (range 65–85) and were followed for 2.8 years (range 1–6) (Table 2).

**Table 2:** Clinical features of peripheral neuropathy associated with wild type transthyretin amyloidosis (ATTRwt) (18, 21–24).

	Our study	(18)	(21)	(22)	(23)	(24)	Cumulative
*N*	18	12	51	16	50	23	170
Mean age (years)/gender (% men)	72.2 /95%	74.1/100%	78.4/95%	74.9/100%	80/92%	76/91%	77.3/91%
Polyneuropathy	11 (61%)	12 (100%)	21 (51%)	7 (44%)	37 (74%)	16 (70%)	104/170 (61%)
Carpal tunnel syndrome	17 (94%)	11 (92%)	36 (71%)	13 (81%)	–	20 (87%)	97/120 (81%)
Spinal stenosis	7 (39%)	8 (67%)	–	3 (19%)	5 (10%)	6 (26%)	29/119 (24%)
Dysautonomia	QSART abnl in 1/4	Mild OH in 2/12	–	OH in 8/16	OH in 0/8	QSART abnl in 6/14	17/54 (31%)

abnl, abnormal; OH, orthostatic hypotension; QSART, quantitative axon reflex sweat testing.

All subjects underwent electrodiagnostic testing, including nerve conduction studies and needle electromyography (EMG/NCS), and 4 subjects underwent autonomic testing. Past medical history also included history of spinal stenosis surgery (*n* = 6), knee replacement (*n* = 6) and hip replacement (*n* = 1). There were 6 patients with renal insufficiency that was not related to amyloidosis. Two patients with IgM gammopathy and inclusion body myositis had nerve and muscle biopsies that did not show nerve or muscle amyloid deposits. Vascular ATTRwt deposits were found incidentally on liver biopsy of the patient who was later found to have inclusion body myositis, but there was no evidence of cardiac amyloidosis on 99 m-technetium pyrophosphate scintigraphy and cardiac MRI. The patient with IgM gammopathy was followed by the lead author for 10 years for paraproteinemic neuropathy associated with anti-myelin associated glycoprotein (MAG) antibodies, prior to the diagnosis of ATTRwt cardiomyopathy.

## 4 Neurologic manifestations of wild type ATTR deposition

### 4.1 Peripheral neuropathy

Peripheral neuropathy is a common neurologic disorder associated with dysfunction of sensory, motor and autonomic nerves. Peripheral neuropathy is often suspected in patients with distal sensory loss and paresthesias (e.g., tingling, burning), but other conditions including spinal stenosis and entrapment neuropathies may present with similar symptoms (39). Other common symptoms include weakness, unsteadiness and dysautonomia (39). On examination, patients might have loss of sensation to different sensory modalities (e.g., vibrations, pinprick, light touch), loss of deep tendon reflexes, weakness (usually worse distally) and lack of coordination. Electrodiagnostic testing with EMG/NCS is often used to characterize peripheral neuropathy and demonstrate features suggestive of axonal loss or demyelination (40). In addition to amyloid cardiomyopathy, amyloid neuropathy is one of cardinal clinical manifestations of systemic involvement in patients with ATTRv (41). Peripheral neuropathy associated with ATTRv is associated with amyloid deposition in peripheral nerves, plexuses and ganglia leading to extensive axonal loss (2, 42). Small cohort studies of ATTRwt patients with cardiomyopathy reported prevalence of peripheral neuropathy ranging widely from 3% to 74% of their patients (23, 43). Clinical significance of ATTRwt neuropathy is still controversial, with its indolent course contrasting the typical progressive clinical course of ATTRv-associated neuropathy and multiple comorbidities. Two considerations confound the association of peripheral neuropathy with ATTRwt. Firstly, in elderly patients with ATTRwt and neuropathy, a nerve biopsy is usually not obtained resulting in the lack of a definitive diagnostic test to distinguish between amyloid polyneuropathy and other etiologies that are prevalent in the same age group (e.g., paraproteinemia, diabetes). Reports of biopsy-proven ATTRwt polyneuropathy are rare (10), but ATTRwt deposits also co-localize with mutant ATTR deposits in peripheral nerves of patients with ATTRv neuropathy (38, 44). Secondly, symptoms of spinal stenosis and chronic radiculopathies may overlap with those of peripheral neuropathy. In this regard, EMG/NCS is useful to characterize large fiber polyneuropathy and distinguish it from radiculopathies and entrapment neuropathies. In our UPMC cohort, electrodiagnostic testing showed axonal sensorimotor polyneuropathy (*n* = 10) and distal sensory polyneuropathy (*n* = 1), while there was no evidence of large fiber peripheral neuropathy in 7 patients. EMG/NCS showed radiculopathies in 7 patients (lumbosacral, *n* = 7; cervical, *n* = 1), with one patient having both cervical and lumbosacral radiculopathies.

### 4.2 Dysautonomia and small fiber neuropathy

Symptoms of dysautonomia, especially orthostatic hypotension, may significantly complicate management of cardiomyopathy and heart failure. Dysautonomia may be associated with sensorimotor peripheral neuropathy involving both large and small nerve fibers or with small fiber neuropathy which involves only thinly myelinated and unmyelinated nerve fibers. Small fiber neuropathy may manifest with numbness, paresthesias and dysautonomia, but deep tendon reflexes, motor strength and coordination are preserved (normal) (45). Conventional electrodiagnostic testing (EMG/NCS) is normal in patients with small fiber neuropathy as it tests only the function of large nerve fibers. Skin biopsy may demonstrate the loss of small nerve fibers, but usually does not reveal the underlying cause (46). Autonomic symptoms with small fiber amyloid neuropathies may include orthostatic hypotension, gastroparesis, diarrhea or constipation, loss of bladder control, erectile dysfunction, loss of sweating with heat intolerance, or arrhythmias (47). Painful sensorimotor polyneuropathy with dysautonomia is a classic manifestation of two most common types of amyloid neuropathies, including ATTRv and light chain amyloidosis (AL) (48), but dysautonomia is generally not considered as a significant manifestation of ATTRwt. Most series reporting ATTRwt-related neuropathy did not find widespread or severe dysautonomia, except for few patients with mild signs of dysautonomia (Table 2) (18, 24). Nevertheless, a recent study by Campagnolo et al. found orthostatic hypotension in 8/16 of their ATTRwt patients, including 5/13 of ATTRwt patients with neuropathy (22). Overall, autonomic testing was abnormal in 31% of ATTRwt patients with suspected neuropathy as reported by different authors (Table 2). In our cohort, autonomic testing (including quantitative sudomotor axon reflex testing and tilt table testing) was performed in only 4 patients, with normal results in 3, and reduced stimulated sweat output without generalized dysautonomia in 1 patient. However, as only 4 of our subjects (22%) underwent autonomic testing, these results may not be representative of ATTRwt as whole. An earlier cohort study of ATTRwt with cardiomyopathy reported dysautonomia in 12% of their patients (43). This may suggest that the prevalence and clinical significance of dysautonomia and small fiber neuropathy may still be underestimated in this population.

### 4.3 Carpal tunnel syndrome

Carpal tunnel syndrome (CTS) is a common condition affecting 4%–8% of general population, and it is often classified as a work-related condition associated with repetitive wrist bending (49). Clinical symptoms usually start with paresthesias and numbness in the thenar and first three digits due to compression of the median nerve at the wrist, later followed by weakness and thenar atrophy. Amyloid deposition in the tenosynovial tissue leading to median nerve compression, often precedes systemic manifestations of different types of amyloidosis including ATTRv, ATTRwt and AL (6, 11, 50). Nevertheless, carpal tunnel syndrome is not a direct manifestation of peripheral nerve amyloidosis as the nerve injury is caused by nerve compression from extraneural tenosynovial amyloidosis. ATTRwt deposits were found in 5% of patients older than 50 (men) or 60 years (women) undergoing carpal tunnel release surgery, and concurrent ATTRwt cardiomyopathy was found in 20% of patients with tenosynovial ATTRwt deposits (6). There may be a prolonged delay of 5–9 years between CTS onset and the diagnosis of cardiac amyloidosis (27). Analogously, cardiology studies reported a history of CTS in 54%–60% of patients with ATTRwt (5, 28). Higher increase of risk for CTS was found in men with both ATTRwt and ATTRv when compared to AL, especially after age of 70 (27). Various studies reported a high prevalence of bilateral CTS with ATTRwt and neuropathic symptoms (Table 2), but bilateral CTS is not specific for amyloidosis as up to 87% of CTS patients have bilateral median nerve entrapments (51). Similarly, an increased risk of CTS was reported with other hereditary and diabetic neuropathies (52, 53).

### 4.4 Spinal stenosis

Spinal stenosis and narrowing of the spinal canal is a common finding in elderly population, typically manifesting with back and extremity discomfort, with some patients having previously undiagnosed cardiac amyloidosis (26). Clinical symptoms typically include back or neck pain, followed by radicular pain of lower and upper extremities and sensory loss due to nerve compression. Radiologic changes of osteoarthritis of spine were reported in up to 66% of individuals older than 65 years (12). Diagnosis is based on physical examination and clinical history and confirmed with imaging studies. Electrodiagnostic testing (EMG/NCS) may demonstrate spinal root impingement (radiculopathies), and severe spinal stenosis may even lead to spinal cord compression and myelopathy. Deposition of ATTRwt amyloid in spinal ligamentum flavum may precipitate spinal stenosis and radiculopathy, or even compressive myelopathy in the absence of amyloidosis of the peripheral nervous system, and ATTRwt deposits were found in 13% of patients undergoing laminectomy. While patients with ATTRv may also have spinal stenosis, it appears more common with ATTRwt, and in a single center study from Germany, 5% of ATTRv and 14% of ATTRwt patients had significant spinal stenosis (28). However, in the same series, ATTRv patients were significantly younger than ATTRwt patients (mean age 59 vs. 74 years) (28). In our series, 39% of ATTRwt patients with suspected peripheral neuropathy had spinal stenosis, compared to 24% from combined series (Table 2). Symptoms of spinal stenosis and radiculopathies can often mimic clinical manifestations of peripheral neuropathy and significant back discomfort is sometimes absent. However, one important distinction is that spinal stenosis and resulting radiculopathies are again a sign of amyloid deposition in the connective tissue and not in the peripheral nervous system, same as with carpal tunnel syndrome and amyloid deposition in the flexor retinaculum.

### 4.5 Musculoskeletal conditions (ligaments, joints and tendons)

Clinical significance of ATTRwt deposition in musculoskeletal and connective tissue is still not well understood. Amyloid deposits associated with osteoarthritis also often contain other types of amyloid including apolipoprotein A-I (13). In addition to spinal ligamentum flavum (26), connective tissue ATTRwt deposits are also found in knee menisci and articular cartilages (13), and rotator cuff tendons (14). Additionally, ATTRwt deposits on biceps brachii tendons may lead to biceps tendon rupture, likely attributable to its reduced elasticity (8). While, distal biceps tendon rupture was reported in up to 1/3 of patients with ATTRwt cardiomyopathy (8), its incidence is far higher before the age of 65 (54), and in younger patients it may not be associated with ATTRwt cardiomyopathy (55). We did not encounter any ATTRwt patients with distal biceps tendon rupture.

### 4.6 Myopathy

Various skeletal muscle disorders (myopathies) typically manifest with muscle weakness and myalgias and may be associated with elevated levels of serum creatine kinase signifying muscle breakdown. In addition to cardiac muscle, peripheral nerves and connective tissue, ATTR deposits may accumulate in skeletal muscle as well, including both ATTRv and ATTRwt deposits (15) We did not find any cases of ATTRwt myopathy in our series, and its prevalence remains unknown with the literature consisting mostly of case reports and small case series. Biphosphonate bone imaging may also reveal ATTRwt skeletal muscle deposits (56, 57). In most of reported cases, ATTRwt myopathy was accompanied with symptoms of neuropathy, but electrodiagnostic testing may be normal (15). Additionally, in ATTRwt myopathy, muscle CK may be normal or slightly elevated, and EMG may show myopathic motor unit potentials without fibrillation potentials (15). Cases of ATTR myopathy mimicking inclusion body myositis were reported for both ATTRv and ATTRwt (58, 59).

### 4.7 Brain/eye

Amyloid TTR deposits can be also found in central nervous system, and transthyretin is produced in the choroid plexus and retina (1). Leptomeningeal ATTR is a rare complication of ATTRv with severe morbidity (60). At this time there are no reports of leptomeningeal ATTRwt, and histopathologic post-mortem analysis of tissue of ATTRv patients after previous liver transplant showed that 39% of ATTR amyloid deposits in the sural nerve belonged to ATTRwt, but in CNS, only 2%–3% of ATTR deposits were wild type (61). While vitreous amyloid deposits have not been reported in patients with ATTRwt cardiomyopathy, vitreous ATTR in patients with heterozygous ATTRv contains both variant and wild type TTR deposits (62).

## 5 Neurologic and other systemic comorbidities

A high prevalence of monoclonal gammopathy was reported in both variant and wild type ATTR (63), and rarely both ATTR and AL may coexist in the same patient (64).

Neuromuscular comorbidities in our series included three cases of inclusion body myositis, primary lateral sclerosis and paraproteinemic polyneuropathy associated with IgM gammopathy and elevated titers of anti-MAG antibodies in one subject each.

## 6 ATTRwt and organ transplantation

As most transthyretin is synthesized in the liver, transplantation of the liver has been used to block the synthesis of the mutant transthyretin in ATTRv. Liver transplantation was the first intervention showing clinical benefits and slowing down ATTRv progression, especially for patients with Val30Met variant (65). However, one of the limiting steps was cardiac accumulation of ATTRwt after transplantation (65, 66). Posttransplant ATTRwt accumulation in the heart and peripheral nerve is likely precipitated by prior amyloid seeding, with histopathologic studies showing the lack of new amyloid deposits in the heart allograft and abundant deposits in the native tissue after heart/liver transplantation in ATTRv (65). Patients with ATTRwt-related heart failure may undergo heart transplantation, and so far there are no reports of ATTRwt cardiac recurrence in these patients (67). There was a report of post-transplant neuropathy and dysautonomia in a patient with ATTRwt, but there was no mention of etiology of neuropathy and dysautonomia (68).

## 7 Discussion

Until recently, ATTRwt was generally considered as an exclusively cardiac condition and was not thought to be associated with peripheral neuropathy or other multisystemic manifestations. However, with the increasing diagnosis of ATTRwt with cardiac scintigraphy and an aging population, there is mounting awareness of the presence of multisystemic symptoms in some patients, including peripheral neuropathy and dysautonomia, complicated by frequent comorbidities. Various clinical studies, including ours, showed large fiber polyneuropathy in ATTRwt patients, and similar symptoms of numbness and dysesthesias may be related to entrapment neuropathies, spinal stenosis and radiculopathies (18, 19, 21–24).

Furthermore, incidental finding of ATTRwt in non-cardiac tissue is not always associated with systemic amyloid deposition and amyloid cardiomyopathy, although the risk of future amyloidosis should be considered. An early single-organ stage of amyloidosis may be followed by systemic spread years later as already demonstrated with carpal tunnel syndrome (50). Possible association of ATTRwt and dysautonomia is intriguing. An increased risk of dysautonomia has been reported in frail elderly individuals, with 29% of population older than 65 reported to have initial orthostatic hypotension (69, 70). Nevertheless, there was no reported male predominance in elderly with dysautonomia as seen with ATTRwt, and the etiology is likely multifactorial in most patients.

At this time, there is a growing evidence of association between ATTRwt cardiomyopathy and ATTRwt deposition in musculoskeletal and connective tissue. In elderly patients, aging is associated with rising prevalence of orthopedic complications (e.g., lumbar stenosis, carpal tunnel syndrome, rotator cuff syndrome) and osteoarthritis, paralleling increasing prevalence of ATTRwt. However, ATTRwt cardiomyopathy is predominantly found in men, and there is no such gender imbalance with the orthopedic complications described above. There is still no convincing evidence that ATTRwt is a major cause of dysautonomia in the elderly population. While increasing prevalence of cardiac ATTRwt with aging may be followed (or accompanied) by a similar process in the peripheral nervous system, there is a discrepancy between the growing number of patients diagnosed with ATTRwt cardiomyopathy and few documented reports of ATTRwt neuropathy with amyloid deposits in the peripheral nerve. Overall, it is still unresolved whether ATTRwt-associated neuropathy is a rare phenomenon affecting few patients, or whether its prevalence and clinical significance are greatly underestimated. Given the advanced age of most ATTRwt patients, a high prevalence of comorbidities is expected, and various symptoms often have multifactorial etiology. Additionally, clinical significance of ATTRwt myopathy appears mostly limited with only rare patients developing clinical symptoms. The presence of amyloid deposition in musculoskeletal and connective tissue may mimic neuromuscular complications and the role of dysautonomia deserves a closer look.

Overall, recent reports of ATTRwt-associated neuropathy stand in contrast to the lack of conclusive evidence of etiology in most cases and paucity of biopsy-proven cases of ATTRwt neuropathy. While the rising incidence of ATTRwt in elderly parallels the incidence of peripheral neuropathy in the aging population, the potential role of ATTRwt in peripheral neuropathy in the elderly and its magnitude are still unclear. Based on currently available information, ATTRwt-associated neuropathy most likely has limited clinical significance for most patients, but additional information may support its greater role. Large longitudinal studies are needed to characterize clinical features and significance of multisystemic involvement including peripheral nervous system complications associated with ATTRwt.
